# Region-Specific Adult Neural Stem Cell Niches of the Mediobasal Hypothalamus and Medulla Oblongata

**DOI:** 10.1007/s12015-025-10995-5

**Published:** 2025-10-21

**Authors:** Eriko Furube, Rena Fujii, Yuri Nambu, Daishi Hiratsuka, Ryoichi Yoshimura, Seiji Miyata

**Affiliations:** 1 Department of Functional Anatomy and Neuroscience, Asahikawa Medical University, Midorigaoka-higashi, Asahikawa, 078-8510 Japan; 2 Department of Applied Biology, Kyoto Institute of Technology, Matsugasaki, Sakyo-ku, Kyoto, 606-8585 Japan; 3 Department of Anatomy and Neuroscience, Osaka Metropolitan University, Asahimachi, Abeno-ku, Osaka, 545-8585 Japan; 4 FUJIFILM Wako Pure Chemical Corporation, Doshomachi, Chuo-Ku, Osaka, 540-8605 Japan

**Keywords:** Brainstem, Mediobasal hypothalamus, Neural stem cells, Neurogenesis, Oligodendrogenesis, Adult brain

## Abstract

The presence of neural stem cells (NSCs) of the subventricular and subgranular zone in the adult mammalian brain has been the focus of much attention; however, these high-function centers have low regenerative ability in response to brain damage. In this review, we focus on the mediobasal hypothalamus (MBH)—a diencephalic region lining the floor of the third ventricle—and the medulla oblongata, a brainstem structure. Both contain niche-like glial populations with context-dependent neurogenic and gliogenic potential. These evolutionarily conserved regions contain neural circuits essential for life support and display high regenerative capacity in lower vertebrates. Recently, NSCs and neural progenitor cells (NPCs) have been reported in the MBH, including the arcuate nucleus and median eminence. Mediobasal hypothalamic tanycytes, with proximal cell bodies facing the third ventricle and distal cellular processes toward the parenchyma, are identified as NSCs that supply various progenitor and ependymal cells. Neural circuits of the MBH exhibit relatively regenerative capability with near-complete or alternative neuronal circuit reorganization after hypothalamic neuronal damage. In the medulla oblongata, there are two types of NSCs: astrocyte-like NSCs in the area postrema and tanycyte-like NSCs in the central canal facing the cerebrospinal fluid. Astrocyte-like NSCs exhibit relatively active proliferation, whereas tanycyte-like NSCs are almost quiescent. Monosodium glutamate selectively induces neuronal cell death in the area postrema, and NPCs proliferate and differentiate into mature neurons, resulting in near-complete restoration of neuronal density. Experimental autoimmune encephalomyelitis causes demyelination in the medulla oblongata, and NSCs partially restore the density of oligodendrocytes. Thus, recent studies indicate that the adult MBH and medulla oblongata exhibit context-dependent regenerative responses, supplying new neurons and oligodendrocytes in response to brain damage.

## The Discovery of Neural Stem Cells (NSCs) in the Adult Mammalian Brain

NSCs can reveal potency for self-renewal and multipotent differentiation to give rise to the three major cell types: neurons, astrocytes, and oligodendrocytes [1, 92]. NSCs exist as neuroepithelial cells around the ventricles, and they repeatedly self-renew through symmetric division without differentiating into neurons or glial cells in the early stages of embryonic brain development, but later they become radial glial cells to produce new neurons in the middle stages [61]. During the neuron-producing period, most NSCs continuously undergo self-renewal and neuronal provision directly or through intermediate progenitor cells [108]. In the late fetal stage, NSCs cease to produce neurons and alternatively generate glial cells such as astrocytes and oligodendrocytes [61].

The Nobel Prize for Physiology and Medicine was awarded to Santiago Ramón y Cajal in 1906 for studies on the nervous system structure or “neuron theory”, which significantly contributed to the history of science. He described that “once the development was ended, the founts of growth and regeneration of the axons and dendrites dried up irrevocably” [38], which means that the brain and spinal cord cannot generate new neurons and glial cells by NSCs and regenerate axons and dendrites based on the regenerative failure of the human brain and spinal cord. Although this dogma has been believed for a long time, in the early 1960 s, Altman and his coworker discovered adult neurogenesis in rodents that the granule cells of the hippocampal dentate gyrus (DG), cerebral cortex, and olfactory bulb (OB) continue to be generated using autoradiography with tritium-labeled thymidine [4, 6]. Most neuroscientists did not accept these challenging findings since there was no concept that NSCs could produce new neurons and glial cells in the adult brain and no adequate methods to identify NSCs, neurons, and glial cells at that time. It has taken approximately one hundred years to establish the existence of NSCs in the adult mammalian brain from the proposal of “neuron theory.”

In the 1990 s, several developments finally established neurogenesis in adult rodents. First, [201] successfully isolated a neurosphere cell aggregate from adult mice that proliferates, giving rise to mature neurons and glial cells. The discovery of neurosphere was historic because it suggests that undifferentiated cell populations, namely NSCs, remain in the adult mouse brain. Second, technical advances in the fate-mapping method using 5-bromo-2’-deoxyuridine (BrdU) in animals have allowed researchers to identify newly generated cells [98]. Several immunohistochemical studies reveal that new neurons are added to the granule cell layer of the DG of adult rodents with these techniques [39, 218].

Nevertheless, NSCs have been considered absent in the human brain because of the preconceived notion that human memory is maintained by synaptic circuitry between neurons, and neurogenesis is dangerous for disrupting synaptic circuits. In 1998, however, [70] demonstrated neurogenesis in the DG of cancer patients who had been given BrdU to detect NSCs using double immunohistochemistry with antibodies against BrdU and the neuronal marker NeuN. The continuous generation of new neurons in the hippocampus is also confirmed in Old-World primates using BrdU immunohistochemistry [129]. In adult mammals, research about NSCs is mainly performed in two brain regions: the subgranular zone (SGZ), which under normal conditions gives rise to interneurons in the hippocampal DG [88, 93], and the subventricular zone (SVZ) around the anterior horn of the lateral ventricle, which produces interneurons in the OB [45, 179, 212].

Long-term running enhances long-term potentiation and promotes neurogenesis in the hippocampal DG of aged mice [245]. Regular aerobic exercise training enhances spatial memory and increases the volume of the anterior hippocampus, with a more pronounced increase in serum brain-derived neurotrophic factor levels in older individuals [69]. On the other hand, stress-induced depression suppresses hippocampal neurogenesis in the adult mouse brain, whereas antidepressants conversely promote neurogenesis [66, 67]. Neurogenesis maintains the memory capacity of the hippocampus in the mouse DG by erasing old memories from the hippocampus and transferring them to the cerebral cortex to prevent saturation of the hippocampal memory capacity [126]. Patients with obesity, diabetes mellitus, hypertension, hypoxic brain injury, bipolar disorder, and head trauma, and aged humans often have smaller hippocampal volumes [79, 223]. Neurogenesis in the adult hippocampus of the rodent brain is essential for normal endocrine system responses and behaviors to stress [225].

Recently, [226] claimed that neurogenesis occurs in young humans until at most 13 years of age, but the rate of neurogenesis declines rapidly with age, and it is rare in adult humans. However, several groups suggest that there is not enough data to deny the concept that adult human neurogenesis contributes to hippocampal plasticity and cognition across the lifespan [31, 125, 162]. These discrepancies may be due to methodological problems such as delays in fixation time, duration of fixation, and variations in expression marker proteins [78]. Recently, single-nucleus RNA sequencing demonstrated the presence of a substantial number of immature neurons in the adult human hippocampus with low-frequency *de novo* generation [268]. However, a simple understanding of the functional significance of adult hippocampal neurogenesis, based on rodent or non-primate studies, needs to be revised for humans.

In the SVZ of adult rodents, type-B NSCs give rise to type-C transient amplifying neuronal progenitor cells (NPCs) that, in turn, give rise to immature or type-A NPCs [8]. The SVZ, which borders the lateral ventricle, provides new neurons for the OB after a long migratory journey along the rostral migratory stream [5]. Continuous neurogenesis in the OB derived from the SVZ is involved in the olfactory behavior of adult mice [212]. In contrast to rodents, robust proliferation and migration of NPCs occur in infant humans before 18 months, while this activity quickly declines in older children and is nearly extinct in adulthood [213]. Moreover, SVZ-derived NPCs migrate into the striatum in adult humans and non-human primates [22, 71]. SVZ-derived neurogenesis is increased in the brains of stroke patients [117] and Huntington’s disease [56]. Although the functions of NSCs in the SVZ of the adult brain are assumed to be vastly different between rodents and humans in considering their migrating routes, there is a commonality concerning the ability to supply new cells in the event of brain injury [27, 71].

## Mammalian Brain Lacks Regenerative Potential?

### Evolution of Adult Neurogenesis in Vertebrates

Adult neurogenesis in the brain is a common phenomenon that has been conserved across vertebrate evolution from fish to amphibians, reptiles, birds, and primates. Adult neurogenesis is likely to decline in terms of the number of neurogenic zones and new neurons added to the adult brain circuits, from fish to mammals [12]. The functions of adult neurogenesis in different species may relate to their regenerative ability, learning, and memories [9]. In adult fish, up to 16 neurogenic zones can be detected, and most proliferating cells are radial glial cells in periventricular zones between the dorsal zone and ventricle wall, and the widespread occurrence of these zones is possibly related to high regeneration capability [215]. In the adult lizard brain, cell proliferation is primarily observed in the ependymal layer of the lateral ventricles [142]. In adult birds, the proliferation of NSCs/NPCs is seen chiefly along the ventrolateral and dorsomedial ventricular wall zones, and these cells are identified as the radial glial cells [7]. Coordinated neurogenesis and angiogenesis occur in the higher vocal center of the adult songbird neostriatum throughout life, depending on gonadal steroids [144]. Adult neurogenesis is present in the dorsal lateral nucleus of the telencephalon of fishes, the dorsomedial cortex of reptiles, and the parahippocampal area in birds that resemble mammalian DG [12]. In mammals, adult neurogenesis is limited to replacing old neurons with new ones, and the areas where neurogenesis occurs are highly restricted [97, 208]. The NSCs in the SGZ of the human brain can generate new neurons throughout adulthood, contributing to brain function [69, 227]. The presence of NSCs is reported in the human striatum [71], lateral ventricular wall [55], and to a limited extent, in the OB [27] of the human brain. These results suggest that neurogenesis has become a limited capability during the evolutionary process, likely due to the increased complexity of brain structure and function. A different possibility is that the excavation of NSCs in adult mammals is incomplete, so we cannot rule out the possibility that they may be present throughout the adult brain and only lack regenerative potential or are quiescent due to some inhibitory mechanisms. Endogenous NSCs in the mammalian brain are activated in response to brain injury and produce new neurons and glia to regenerate damaged brain tissue; the injury-induced endogenous neurogenesis is inadequate for repairing impaired neural function. Scientists have attempted to enhance endogenous neurogenesis using various strategies, including using neurotrophic factors, bioactive materials, cell reprogramming, and pharmaceutical tools to manipulate endogenous NSCs to reach practical benefits for the treatment of brain diseases [14, 127, 172].

### Limitation of Regenerative Capacity of Mammalian Higher Function Centers

Higher function centers of the adult brain, such as the neocortex and hippocampus, exhibit neuronal cell loss and degeneration due to direct physical damage and exposure to excitatory neurotransmitter glutamate following traumatic injury, ischemia, and stroke [199]. In the adult brain SGZ, mild traumatic brain injury facilitates the proliferation of NSCs and NPCs and a subpopulation of these cells differentiating into mature neurons in the granule cell layer, but the supply of new neurons from NPCs declines accompanied by an increase in mature granule cells [180, 229]. After severe traumatic brain injury, however, most NPCs migrate to misplace at the outer layer of the hippocampus across the border from the inner granular cell layer [112]. Thus, NPCs appear to enact near-complete regeneration of neural tissue only in the case of mild damage, with an expected restoration of short-term memory. In the SVZ, NPCs migrate toward the adult mouse striatum and neocortex after cerebral ischemia through scaffold cells, including astrocytes and blood vessels [43, 173]. The SVZ-derived NPCs migrate to the damaged area and then survive at least 28 weeks after the elimination of neocortical neurons [145]. However, their migration is inconsistent, unlikely to reach the cerebral cortex lesion site, and cannot survive without successful maturation and integration into the local neural network [122]. The low regenerative ability of these higher-function centers may be a strategy to prevent memory malfunction in higher-order brain functions. Consistent with this, in Alzheimer’s disease the neurogenic response is strongly attenuated despite ongoing degeneration [187, 270]. In addition, an inflammation-induced growth-inhibitory niche characterized by glial-scar-associated extracellular matrix and pro-inflammatory cytokines (e.g., interleukin (IL)−1β, tumor necrosis factor-α, IL-6, interferon-γ) acting via nuclear factor-kappa B and Janus kinase/signal transducer and activator of transcription pathways can further suppress NSC/NPC proliferation and neuronal differentiation [23, 54, 207, 209].

## The Mediobasal Hypothalamus (MBH) Is a New Source of Neurons and Glial Cells

### NSCs/NPCs in the MBH

The functional significance of NSCs in the adult mammalian brain is region-specific, so exploring the presence of NSCs in brain areas other than the SGZ and SVZ is important. It has recently been shown in the adult mammalian brain that NSCs exist in brain regions other than classical neurogenic niches mentioned above and give rise to neural and glial cells (See detailed reviews: [29, 121]. The structural and functional organization of the adult MBH and the brainstem medulla is remarkably conserved across vertebrate species, reflecting their shared roles in core homeostatic control [146, 254]. This remarkable conservation may correspond to conserved molecular mechanisms that regulate neurogenesis [146, 254]. In the hypothalamus, proliferating cells with a stellate shape are obtained from neurosphere cultures of hypothalamic tissues around the third ventricle in at least 15-week-old adult rats [72]. Similarly, neurosphere cells derived from a 7-week-old rat hypothalamus can differentiate into mature neurons expressing various adenohypophysis-releasing hormones, somatostatin, oxytocin, and vasopressin [149]. The number of BrdU-labeled cells in the arcuate nucleus (Arc) is enhanced by fibroblast growth factor-2 (FGF-2) in vivo [256]. Intracerebroventricular infusion of the ciliary neurotrophic factor induces the generation of new neurons in the Arc and long-term weight loss in the adult mice via leptin-like pathways, while inhibition of cell division in the Arc attenuates ciliary neurotrophic factor-induced weight decrease [128]. The median eminence (ME) is one of the brain regions of the circumventricular organs (CVOs) and possesses fenestrated capillaries [158–160]. Doublecortin (DCX)-expressing cells are observed in ventral regions of the Arc of the adult human brain [20]. Nestin- and polysialylated-neural cell adhesion molecule-expressing cells are present, and Ki-67-expressing proliferative cells are increased by stroke in the ME of adult humans [214]. Thus, NSCs/NPCs in the MBH can produce new neurons and glial cells in adult humans and rodents. Consistent with these histological observations, brief queries of the Allen Human Brain Atlas and the Human Brain Transcriptome show detectable expression of NSC/gliogenic markers such as SRY-box transcription factor 2 (SOX2), nuclear export signal, and vimentin and oligodendrocyte lineage markers such as oligodendrocyte transcription factor 2, platelet-derived growth factor receptor α, and SOX10 in adult hypothalamus and medulla/pons. By contrast, neuroblast markers such as DCX and achaete-scute homolog 1 remain low outside the hippocampus [102, 123].

#### Methodological Considerations and Limitations

Much of the evidence summarized here relies on neurosphere assays, BrdU birth-dating, and injury paradigms. Neurosphere formation indicates the presence of mitotically competent precursors under mitogen-rich conditions but does not, by itself, establish stemness, clonal self-renewal at limiting dilution, or in vivo lineage potential; serial passaging can bias toward highly proliferative clones and against quiescent NSCs [201]. BrdU incorporation reports S-phase, but it can label proliferating glia or endothelial cells and, after injury, cells undergoing DNA repair; pulse-chase results depend on dose, clearance, and antigen retrieval, and label dilution can obscure long-term fates [98]. Inducible genetic lineage tracing (e.g., Nestin-, glial fibrillary acidic protein (GFAP)-, Rax-, or Foxj1-CreER reporters) mitigates some of these issues but remains constrained by promoter specificity and recombination efficiency [114]. Acute manipulations with monosodium glutamate (MSG), experimental autoimmune encephalomyelitis (EAE), and irradiation reshape the niche by altering cytokines and vascular access, which can confound interpretation of intrinsic NSC behavior; sex, age, strain, diet, and circadian state further modulate proliferation and fate. These considerations argue for convergent evidence across assays and standardized reporting of dosing, chase intervals, and clonal analyses when inferring adult neurogenesis or gliogenesis.

### α- and β-tanycytes

[109, 203–205]. Tanycytes have been classified into α1, α2, β1, and β2 subtypes based on ventricular location, protein expression, ultrastructure, and proliferative activity [2, 203, 204, 271]. β-tanycytes line the infundibulum and ME of the MBH, whereas α-tanycytes are present on the slightly dorsal side of the third ventricular zone adjacent to the Arc and ventromedial nuclei [203–205]. α2-tanycytes extend their cellular processes to the Arc, approximately 300 μm in width, and make contact with the blood-brain barrier (BBB)-containing capillaries of the Arc [203].

α-tanycytes can convert CSF glucose to lactate depending on the connexin-43 gap junction, which transmits to proopiomelanocortin neurons in the Arc via monocarboxylate transporters to sustain neuronal activity [138]. Moreover, it is shown that selective suppression of either tanycytic monocarboxylate transporters or gap junctions results in an increase in food intake, fat mass, body weight, and respiratory exchange ratio and vice versa, a decrease in ambulatory activity, oxygen consumption, and energy expenditure [138]. Application of lactate to α-tanycytes drives neuronal activity in proopiomelanocortin neurons, and blockade of lactate synthesis in α-tanycytes reduces neuronal activity and increases mouse body weight and feeding [138]. The direct activation of extrahypothalamic glutamatergic synapses on tanycytes modulates the activity of orexigenic or agouti-related protein (AgRP)-expressing neurons via vascular endothelial growth factor (VEGF) and thereby reduces food intake [25]. Tanycytes can activate ex vivo orexigenic and anorexigenic (proopiomelanocortin) neurons via an ATP-dependent mechanism and trigger acute hyperphagia only in the fed state during the inactive phase of the light-dark cycle [30].

Genetic ablation of β-tanycytes and ventral α2-tanycytes in the ME and Arc decreases tight junction protein ZO-1, disrupts ventricular-hypothalamic barriers, and lowers insulin sensitivity, which increases adiposity under thermoneutral conditions [258]. Both α- and β-tanycytes express functional leptin receptors and have the ability for the transcytotic transport of peripheral leptin into the cerebrospinal fluid (CSF) depending on extracellular regulated kinase-signaling [16, 65]. Selective deletion of tanycytic leptin receptor results in hyperlipidemia and lipid accumulation in white adipocytes, and tanycyte-transported leptin is crucial for the control of β-cell function in the pancreas, probably via the control of leptin-sensing Arc neurons to the pancreas [65].

### Tanycyte of the Mediobasal Hypothalamus Has Characteristics of NSCs

The NSCs in the SVZ have a multi-ciliated apical ending at the ventricular surface which constitutes pinwheel architecture of the ventricular surface, suggesting that they possess fundamental properties of epithelial cells [156]. Tanycytes in the mediobasal hypothalamus have important functions such as control of body weight and feeding behavior, insulin sensitivity, and visceral adipose volume [53]. The question arises as to why new neurons and glial cells are necessary for maintaining and activating various homeostatic functions in adult mammals.

During hypothalamic development, β-tanycytes proliferate during E11 ~ 18 with a peak at E12 ~ 13, whereas α-tanycytes emerge later in development since their cell division is observed during E12 ~ P7 with a peak at E14 ~ 17 [143]. Neurosphere cells from the brainstem of 15-week-old rats reveal proliferative potential and expression of immature and/or mature neuronal marker proteins when plated on a coated culture dish after a passage [72]. NSC/NPC-like cells harvested from the hypothalamus of 7-week-old rats are maintained even after four passages and display several phenotypes, including GABAergic, dopaminergic, and cholinergic lineages under differentiation conditions [149].

β-tanycytes of infant and young adult mice show a generation of new neurons to the ME, and the selective X-ray irradiation of the ME decreases the number of newly-born neurons [136]. Moreover, in adolescence, mice fed a high-fat diet (HFD) exhibit increased neurogenesis and display significantly lower weight and fat mass when grown into adulthood [96, 136]. However, when mice receive chronic HFD, they reveal the generation of fewer new neurons with an increase in apoptosis of newborn neurons, which lowers the replacement of old neurons [152]. Neurogenesis in the ME is highly increased in female mice rather than male animals when chronically fed on HFD [137]. Lately, however, pharmacological and genetic ablation of NG2-glia in the adult ME has led to obesity with loss of leptin responsiveness, suggesting that weight gain induction caused by X-irradiation in the ME may be due to the loss of oligodendrocyte progenitor cells (OPCs) following irradiation [60]. Stage-specific lineage tracing of FGF-10-expressing β-tanycytes using its transgenic mice reveals neurogenesis at postnatal day 28, lasting as late as postnatal day 60 [100]. FGF-10-expressing β-tanycytes generate orexin- and neuropeptide-Y-expressing neurons mainly in the Arc and are concerned with appetite and energy balance [100]. The MSG treatment selectively eliminates AgRP neurons and induces tanycyte proliferation in the adult mouse Arc, while proliferating tanycyte do not become mature AgRP neurons and a subpopulation of progenitor cells supplies AgRP neurons to recover the number of them within several weeks [261].

β-tanycytes, expressing retina and anterior neural fold homeobox transcription factor, are primarily quiescent, and the differentiation of β-tanycytes into other cell types is limited in adult mice [168]. However, they actively proliferate in an insulin-like growth factor-dependent manner following mechanical injury to the ME [168]. The point mutation of v-raf murine sarcoma viral oncogene homolog B (Braf) is responsible for developing pituitary tumors and adamantinomatous craniopharyngioma via paracrine mechanisms in mice and humans [89]. Craniopharyngioma induction of Braf mutation into β-tanycytes enhances the expansion of tanycytes without differentiation into neurons, and astrocytes leading to the formation of craniopharyngioma-like tumors, suggesting that β-tanycytes are the cell type of origin for craniopharyngioma [168]. Dysfunction of the hypothalamo-neurohypophysial system, reported in the central diabetes insipidus and neuroendocrine hormone disorders, results in abnormal regulation of body fluid metabolism [76]. Recently, however, NPCs in the adult mouse ME have been shown to generate new neurons to reconstruct a new neurohypophysis-like structure for regulating the release of neurohypophysial hormones and restoring the metabolic function of body fluid after the pituitary stalk dissection [185]. GFAP-expressing α2-tanycytes in the Arc possess stem-like neurospherogenic activity, and their proliferation is regulated by FGF-10 and FGF-19 [271]. Estrogen receptor α-expressing neurons are newly generated in the Arc and ventromedial nucleus of the hypothalamus, and a high-fat diet increases the number of estrogen receptor α-expressing neurons [28]. Newly proliferating NG2-expressing cells in the ME differentiate toward mature oligodendrocytes in vitro and are increased by experimental autoimmune encephalomyelitis [269].

### Neurogenic Niche of Tanycytes of the Adult Human Mediobasal Hypothalamus

Human embryonic tanycytes exhibit similarities in gene expression and transcription factor activities to those of mouse tanycytes [15]. By contrast, Nestin- and Sox2-expressing tanycytes or NSCs are localized only at the floor wall of the 3rd ventricle in humans, whose distribution corresponds to that of β tanycytes in rodents [146, 192, 219]. In the adult human hypothalamus, DCX-expressing NPCs are rounded in shape in the ependymal/subependymal zone of the ME and Arc, but fusiform in the Arc and bipolar in the ventromedial hypothalamus [20, 189]. The density of DCX-expressing NPCs is higher in the mediobasal hypothalamus of adult human and sheep brains compared with that of the adult mouse [20]. In humans, a new ectopic posterior pituitary appears in the region of the infundibular recess of the third ventricle in patients with pituitary stalk interruption syndrome [83, 246, 248]. These results tanycytes are identified as NSCs in the adult human and rodent hypothalamus, but there is a difference between humans and rodents.

### Secretome from NSCs of the Mediobasal Hypothalamus

Apart from neurogenesis, it has been demonstrated in the adult brain that morphogens, growth factors, neurotrophins, and cytokines regulate the proliferation and maintenance of NSCs and NPCs, as well as their differentiation, through autocrine and/or paracrine mechanisms (Dause et al., 2022a, 58; [146]. Moreover, hypothalamic NSCs/NPCs are primarily responsible for the secretion of exosomal microRNAs in the cerebrospinal fluid, and these microRNAs in the CSF and tanycyte number are declined with aging, whereas central infusion of hypothalamic NSCs/NPCs-secreted exosomes to the CSF and implantation of a line of newborn mice-derived hypothalamic NSC led to the slowing of aging [266]. It is shown that expression levels of NSC-derived factors such as Wnt and VEGF decline remarkably in the neurogenic niche with aging [187]. Considering the potent role of the NSC-derived secretome in maintaining NSC pools during young adulthood, exhaustion of NSCs and decline of neurogenesis in the aged brain are likely caused or exacerbated, at least in part, by changes in the NSC secretome [266].

### Neuronal Regeneration of the Adult Mediobasal Hypothalamus

**Fig. 1:**
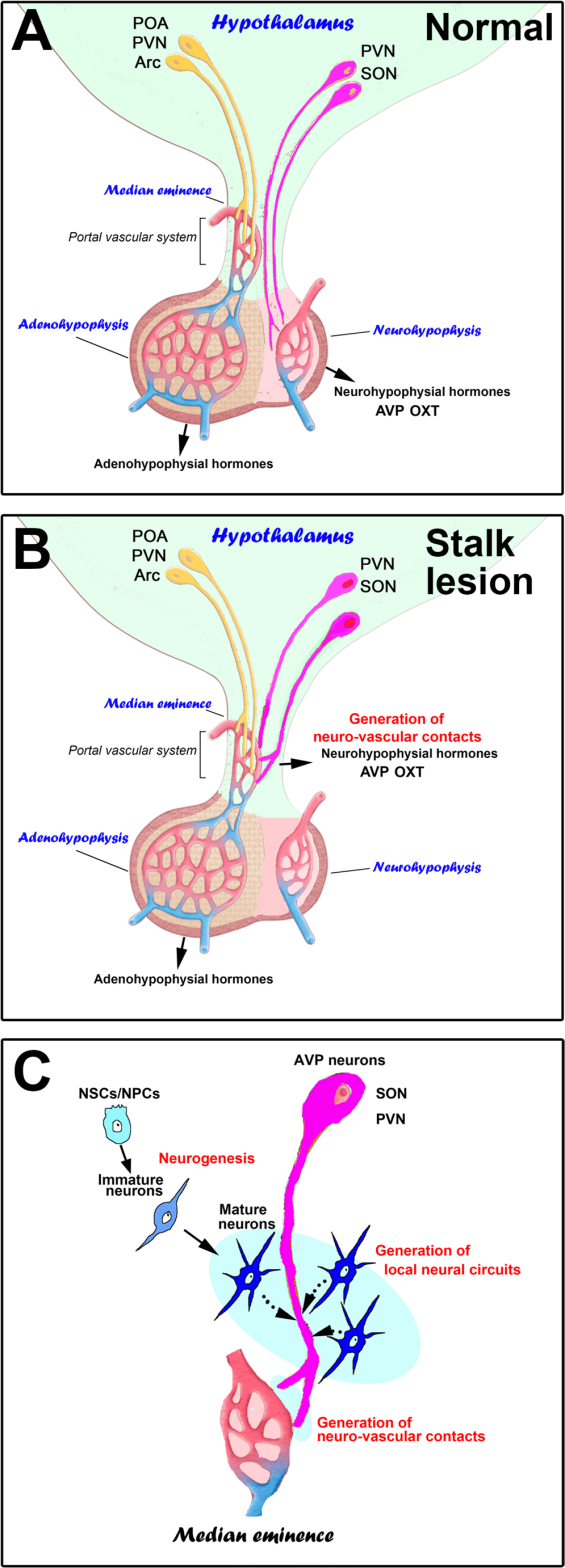
Schematic illustrations of compensatory remodeling of the hypothalamo–hypophyseal axis after stalk lesion: Under normal conditions, magnocellular neurons in the PVN/SON project to the neurohypophysis to release AVP/OXT, while parvocellular neurons terminate in the ME to regulate adenohypophyseal secretion via the portal system (**A**). After stalk disconnection, surviving neurosecretory axons sprout within/around the ME and form de novo neuro-vascular contacts that enable AVP/OXT release into the portal vasculature (**B**). Proliferating NSC/NPCs differentiate into mature neurons, which then interact with vasopressinergic fibers to reconstruct the new local circuit at the fenestrated capillary in the adult ME after the lesion (**C**)

In the adult mouse Arc, agouti-related protein-expressing neurons are newly supplied from post-mitotic NPCs after MSG-induced loss of agouti-related protein-expressing neurons to maintain food and energy homeostasis [261]. Although MSG treatment increases the proliferation of tanycytic NSCs in the Arc, the proliferated tanycytic NSCs are not responsible for replenishing agouti-related protein neurons [261]. Interestingly, post-mitotic progenitor cells differentiate into mature agouti-related protein-expressing neurons [261]. Lesions in the hypothalamo-neurohypophysial system can lead to dysfunction in body fluid metabolic regulation, which leads to central diabetes insipidus and neuroendocrine hormone disorders [11]. However, clinical and animal studies reveal that compensation and recovery of fluid metabolic function happen after hypothalamic injury, suggesting the possibility of reconstructing a new neurohypophysis-like structure [17, 76]. Recently, it has been shown that the repair and reconstruction of a new neurohypophysis-like structure in the ME of adult rats after an electrical lesion of the pituitary stalk [185] (Fig. 1). Proliferating NPCs differentiate into new mature neurons and then integrate to reconstruct the local vasopressinergic neural circuit in the ME after the lesions [185]. Moreover, the transcription factor of NK2 homeobox 1 and the sonic hedgehog signaling pathway are shown to be the possible key regulators of adult hypothalamic neurogenesis during these processes [185]. These results suggest that complete and/or near-complete neuronal regeneration occurs in the hypothalamus to maintain the essential survival functions or the homeostatic regulatory mechanism. Full regeneration does not cause functional malfunction of the neuronal circuits; although MBH circuits include stereotyped homeostatic motifs, they retain substantial plasticity (synaptic, neurogenic, and gliogenic) that supports the restoration of function without destabilizing the mnemonic circuits of higher centers.

## New Cell Sources in the Adult Medulla Oblongata

### Functional importance of the Medulla Oblongata

The medulla oblongata is the lowest part of the brainstem and is connected posteriorly with the spinal cord. The medulla oblongata plays a crucial role in relaying information between the spinal cord and higher parts of the brain, integrating blood-borne, central, and vagal ascending signals, and controlling autonomic activities in cardiovascular and respiratory systems. Additionally, medullary neuronal nuclei regulate various functions essential for maintaining body homeostasis and metabolism [34]. Among the medulla oblongata, the dorsal vagal complex (DVC) consisting of the area postrema (AP), solitary nucleus (Sol), and the dorsal motor nucleus of the vagus nerve, provides the major integrative center for the mammalian autonomic nervous system [35, 242]. In recent years, the DVC has been the focus of considerable attention as discoveries of new functional importance have been reported. For example, activation of pituitary adenylate cyclase-activating polypeptide-expressing neurons induces sickness responses such as a decrease in food and water intake, locomotor activity, and temperature, and inhibition of the neuronal activity significantly attenuates these sickness responses except for the temperature using several transgenic mouse lines [113]. The central melanocortin system of the AP and Sol possesses orexigenic or AgRP neurons to control feeding behavior independently of hypothalamic AgRP neurons [13]. Although the mediobasal hypothalamus is a well-known brain region that senses alterations in circulating hormones and nutrients to maintain whole-body metabolic homeostasis, both the AP and Sol have a similar function by sensing glucose through both glucose transporter 1 and pyruvate kinase [35]. By combining single-cell RNA sequencing, glucagon-like peptide-1 receptor-expressing neurons in the AP may mediate stereotyped behavioral responses to multiple nausea-inducing stimuli [262]. Moreover, it has been shown that glucose insulinotropic peptide, or a gut hormone, activates GABAergic neurons in the AP, which inhibits nausea-promoting excitatory neurons [262]. Blood interleukin-6 can rapidly enter the AP to activate a subpopulation of the AP neurons and its associated network, and the silencing of interleukin-6-induced neuronal activation attenuates cachexia and hyperactivity to prolong the lifespan of tumor-bearing mice [230]. Neuromyelitis optica spectrum disorder is an inflammatory autoimmune disease caused by anti-aquaporin-4 autoantibody with a high risk of recurrence and disability, and the AP is one of the commonly involved brain areas of this disorder [157, 265]. Thus, recent topics indicate that the AP is crucial for feeding and energy homeostasis and pathological responses such as pathogen-induced sickness responses, nausea, cancer cachexia, and neuromyelitis optica spectrum disorder.

### AP Belongs To the Sensory CVOs

The BBB prevents access to blood-derived substances [57, 68], and dysfunctions of the BBB result in the diffusion of various blood-derived substances into the brain parenchyma and subsequent neuronal damage [270]. The CVOs were initially described in 1958 by Austrian anatomist Helmut O. Hofer concerning brain midline structures around the third and fourth ventricles that can contact with blood circulation and cerebrospinal fluid [107]. The organum vasculosum of the lamina terminalis (OVLT), subfornical organ (SFO), ME, AP, neurohypophysis, and pineal gland are collectively referred to as the CVOs and characterized by a highly permeable vascular system, unlike the rest of the brain, which has a BBB-containing vascular system [158–160]. Therefore, the CVOs are also named as the “windows of the brain” [99].

The CVOs are divided into two groups based on their primary functions: the sensory CVOs comprise the OVLT, SFO, and AP, while the secretory CVOs include the ME, neurohypophysis, and pineal gland [158–161]. The vasculature of the CVOs lacks the BBB, and therefore, their vasculature allows parenchyma cells to sense many blood-derived substances, integrate blood-derived information, and transmit it to related brain regions in controlling autonomic, hormonal, and behavioral reactions [158–160]. Interestingly, parenchyma cells including astrocyte- and tanycytes-like NSCs in the CVOs, express several proteins with sensor functions similar to those present in peripheral sensory tissues or cells: such as toll-like receptor 2 and 4 for the innate receptors of pathogen-derived molecules [170, 171, 175] 236; [234], transient receptor potential cation channel subfamily V member 1 (TRPV1) as thermal and mechanosensor [147], Nax as Na^+^ sensor [249], with-no-lysine kinase 1 as osmosensor [118], GPR4 T-cell death-associated gene 8 as proton sensor [231, 253], glucose transporter 1 and pyruvate kinase as glucose sensor [35, 47]. Moreover, a wide variety of humoral and cytokine and their receptors are highly expressed in the CVOs; receptors for vasopressin, purines, glucocorticoid, prostaglandin 2, angiotensin II (McKinley et al., 150; [222], cytokines [151], ghrelin, leptin, and adiponectin [80, 81].

The CVOs are important brain regions for initiation of neuroinflammatory response, for circulating pathogen-associated molecular patterns and/or cytokines to induce faster transcriptional activation of genes encoding for proinflammatory molecules [170, 171, 175, 183, 196, 209, 259]. The CVOs have been considered to function in pathological conditions such as sepsis, stress, the neuronal invasion of parasites, autoimmune encephalitis, and systemic amyloidosis [132, 222], as they are involved in most autonomic and endocrine regulatory pathways. The secretory CVOs, comprising of the neurohypophysis and ME, release various hypothalamus-synthesized neuropeptides [159, 161]. The detailed function of the secretory CVOs is described in the previous review [159].

### NSCs in the Adult Medulla Oblongata

BrdU-labeled nuclei are detected in HuC/D or NeuN-expressing mature neurons and Nestin-expressing cells in the AP of the adult rat brain [21]. Nestin-, GFAP-, and Vimentin-expressing NSCs are present in the adult CVOs using Nestin-EGFP transgenic mice and exhibit proliferative activity and differentiation into neurons and glial cells in vivo and in vitro [26, 46]. Math-1 and Mash1-expressing NPCs are seen to localize close to the fenestrate capillary and express plasminogen, an extracellular matrix-degrading enzyme [111]. Recently, we found that tanycytes in the CVOs and central canal (CC) of adult mice expressed Nestin, GFAP, and Sox2, and their proliferation was increased by chronic intracerebroventricular infusion of FGF-2 and epidermal growth factor (EGF) [84, 86]. Tanycyte-like NSCs exist continuously along the CC from the medulla oblongata to spinal cord using Nestin-CreERT2/CAG-CAT^loxp/loxp^-EGFP mice [84]. Complete separation of AP parenchyma and ventricle-facing tanycyte in neurosphere culture confirmed the presence of tanycyte- and astrocyte-like NSCs in the medulla oblongata of adult mouse brain [177]. Thus, there are two types of NSCs in the CVOs: astrocyte- and tanycyte-like NSCs.

### Astrocyte-like NSCs

**Fig. 2:**
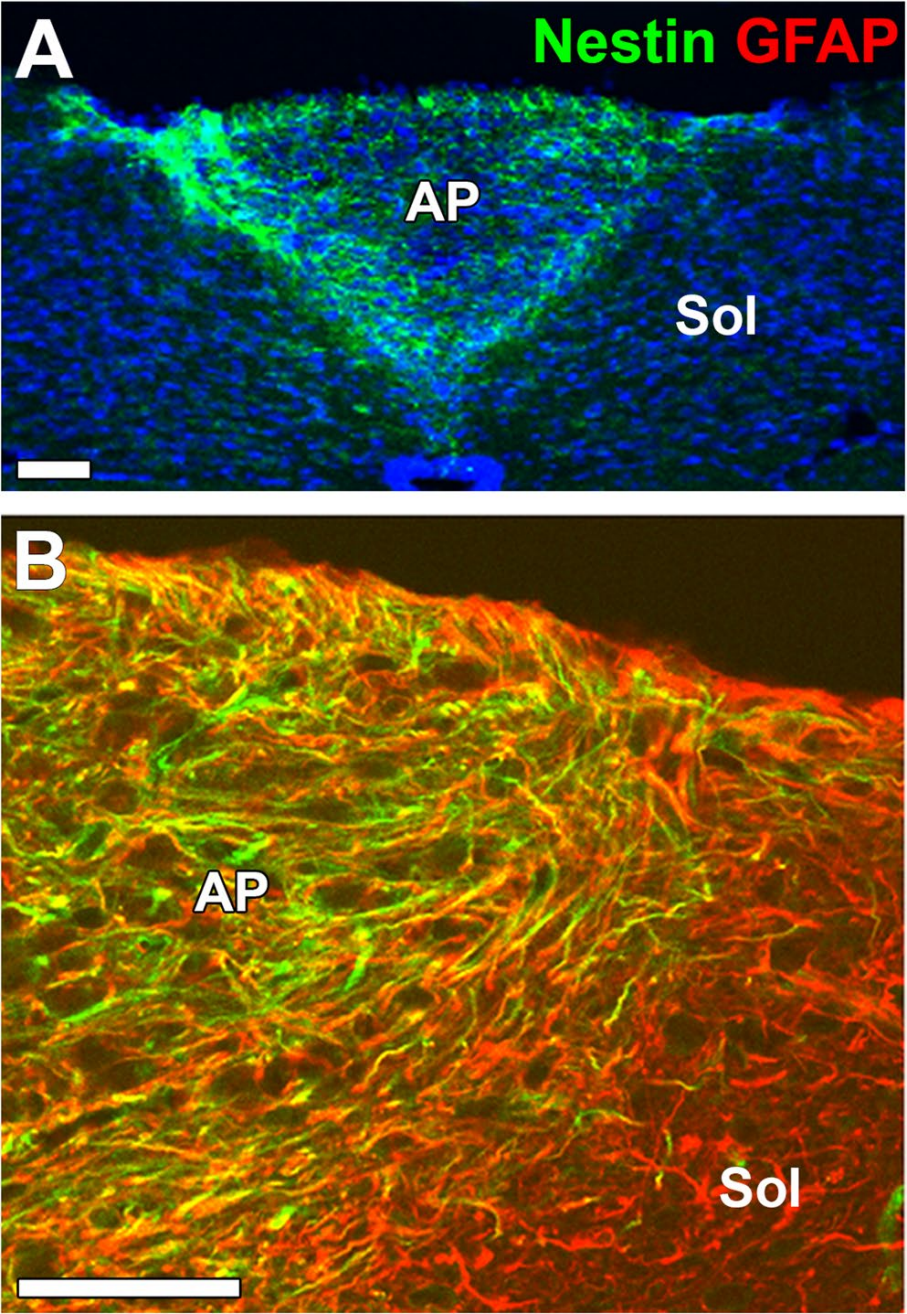
Immunohistochemistry showing specific localization of Nestin-expressing astrocyte-like NSCs in the adult mouse AP (**A**). GFAP-expressing astrocyte-like NSCs express Nestin in the AP, but GFAP-expressing astrocytes do not express Nestin in the Sol (**B**). Scale bars = 50 μm. Photographs are rearranged with permission from Springer Nature [86]

GFAP-, Nestin-, Vimentin-, and polysialylated-neural cell adhesion molecule-expressing astrocytes show a proliferative marker Ki-67 in the CVOs such as the AP, ME, pineal glands, and neurohypophysis of human patients with cerebral ischemia [214]. In addition, astrocytes of human and rodent CVOs express intermediate filament proteins such as Nestin and the transcription factor Sox2 like the common astrocyte markers GFAP, Vimentin, and S100B [26, 84, 86, 111, 214] (Fig. 2). Astrocytes of the adult AP are reported to express Nestin using GFP-fused Nestin or Nestin-CreERT2/GAG-CAT^loxP/loxP^ mice [26, 84, 86]. Furthermore, astrocytes in the AP show BrdU incorporation and Ki67 expression, indicating their proliferative potential [26, 84, 86]. When AP tissues were cultured without tanycytes using the neurosphere assay, they showed proliferative potential even after several passages [177]. These results indicate in the adult AP that astrocytes exclusively have characteristics of NSCs, and there is no typical non-dividing astrocyte.

**Fig. 3:**
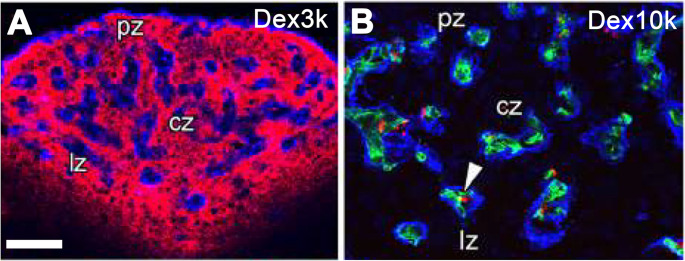
Vascular permeability of dextran 3k and 10k at the AP of the adult mouse brain. Strong fluorescence of blood-derived dextran 3k is seen at parenchyma of the central, perivascular, and lateral zones of the AP (**A**,** B**). In contrast, the fluorescence of dextran 10k is observed only at perivascular space between the inner and outer basement membranes (**C.D**). Asterisks reveal vascular lumen of fenestrated capillary. Dextran 3k, Dex3k; Dextran 10k, Dex10k. Asterisks reveal vascular lumen of complex fenestrate capillary. Scale bar = 50 (A) and 10 (**C**) µm. Photographs are rearranged with permission from Springer Nature [165]

The vasculature of general adult brains possesses the BBB, which consists of an endothelial cellular sheet endowed with tight junctions, prevents free entry of water-soluble low-molecular-weight molecules into the brain parenchyma [267]. The CVOs including the AP, however, possess no typical vascular BBB, and therefore allow blood-derived low-molecular-weight (less than 5000 ~ 10,000) molecules to enter the brain parenchyma [158–160, 166, 167] (Fig. 3). However, the CVOs have a neuronal defense mechanism against blood-derived toxic molecules instead of the vascular BBB, i.e., the cytoplasmic processes of the astrocytes tightly surround neuronal somata [165] and the plasma membranes of the astrocytes are tightly adherent to each other [165]. Thus, astrocytes inhibit low-molecular-weight molecules derived from the fenestrated capillaries to act on the neurons in the AP and to diffuse into the surrounding neuronal nuclei despite the absence of a vascular BBB [158–160, 165]. However, this evidence indicates that any high-molecular-weight molecules cannot penetrate the vasculature of the AP: even in the BBB-containing capillaries, high-molecular-weight molecules, such as various cytokines and growth factors, translocate from blood circulation to brain parenchyma via BBB-specific transcytosis system [257]. In the AP of the adult brain, any high-molecular-weight molecules derived from blood reach perivascular space between inner and outer basement membranes [166, 178], and some of them may move into parenchyma via transcytosis system. Vascular perivascular macrophages sense peripheral signals in the circulation and translate their information into brain parenchymal cells to control hypothalamic-pituitary axis and energy homeostasis [74, 154, 260].

Interestingly, the interaction between NSCs and fenestrate capillary has also been reported in the SVZ: type B NSCs contact on fenestrate capillary, which lacks the tight junctions, astroglial endfoots, and pericytic coverage [220, 238]. This specialized interaction enables NSCs to respond to signals in the perivascular extracellular matrix, hormones, or growth factors in the blood circulation [156]. Contact-independent signaling between NSCs and endothelial cells regulate NSC survival, proliferation and differentiation via pigment epithelium-derived factor, betacelluline, VEGF, and angiopoietin-1 [146]. Moreover, contact- dependent Notch signaling is an important molecular regulator of neurogenesis in the SVZ [95, 146, 192]. In the AP of the adult mouse, astrocyte-like NSCs and neurons express high levels of VEGF mRNA and protein [164]. In vitro co-culture of NSCs and endothelial cells produces a neurovascular environment possibly via autocrine/paracrine and juxtacrine signaling, suggesting that interdependent signaling between NSCs and endothelial cells promotes angiogenesis [51]. Self-renewal and differentiation of hematopoietic stem and progenitor cells toward their mature progeny in the adult bone marrow is tightly regulated by cues from the microenvironment and endothelial cell-derived signals have been shown to regulate their maintenance [272]. Distinct concentrations of radial glia-derived vascular cell adhesion molecule 1 around the ventricular zone of blood vessels and the perineural vascular plexus differently organize cortical angiogenesis during late embryogenesis [264]. Thus, astrocyte-like NSCs in the adult AP may have direct contact with the circulation of the fenestrate capillary to control the proliferation in response to humoral factors.

Testosterone-induced coordinate regulation of neurogenesis and angiogenesis occurs for structure reconstruction via VEGF signaling in the vocal control nucleus of adult female canaries [144]. Cultured NSCs/NPCs support capillary morphogenesis and protect endothelial cell death by hypoxia-inducible factor 1α and VEGF in mouse [206] and human [273]. The overexpression of VEGF prominently promotes angiogenesis and neurogenesis in the adult SGZ, whereas the inhibition of VEGF signaling attenuates exercise-induced increases of vascular density and learning acquisition [140, 241]. Dense clusters of NSCs/NPCs are associated with the vasculature, and some endothelial cells can proliferate in the SGZ [186]. Filopodia of endothelial cells sprout from existing thick microvessels in the molecular layer and dentate hilus of the DG, and treatment of fluoxetine, an antidepressant drug, significantly increases vascular density by enlarging the microvessel luminal size and eliminating the endothelial filopodia, and the number of proliferating NSCs/NPCs and endothelial cells [148]. In the adult mouse AP, the dramatic proliferation of endothelial cells occurs even in normal conditions [165]. AZD2171, a VEGF receptor-associated tyrosine kinase inhibitor, decreases the number of proliferating endothelial cells and filopodia, whereas it decreases vascular density [165]. Brain infusion of AraC, a mitotic inhibitor, decreases the vascular permeability of low-molecular-weight molecules along with the proliferation inhibition of endothelial cells and NSPCs [165, 167]. These results suggest that coordinated regulation of proliferation and differentiation of NSCs/NPCs and angiogenesis occurs in the AP of the adult mouse.

### Tanycyte-like NSCs

As described before, it is established that tanycytes are NSCs in the mediobasal hypothalamus of adult brains. Tanycyte-like ependymal cells are also known to exist in the OVLT and SFO, and CC of the medulla oblongata and spinal cord [158, 160, 203, 205]. Tanycyte-like ependymal cells in the CC also extend long cellular projections into the parenchyma and can transport macromolecules by transcytosis like the mediobasal hypothalamus [182, 203, 205]. These proliferative tanycyte-like ependymal cells exhibit NSC-associated protein expression such as Nestin, GFAP, and Pax6 [85, 177]. Thus, tanycyte-like NSCs are present in the CC of the adult medulla oblongata, like astrocyte-like ones.

In vitro, NSCs generate neurospheres that are clonal aggregates of cells and differentiate into neurons, astrocytes, and oligodendrocytes; therefore, the neurosphere assay helps to find a new neurogenic niche in the adult brain [201]. Neurosphere-generating activity is reported in primary cultures from various locations of the cerebrospinal ventricular walls in addition to the SGZ and SVZ [250]. In addition to the mediobasal hypothalamus, tanycyte-like ependymal cells exist in the CVOs [135] and are devoid of cilia but possess long processes unlike multi-ciliated ependymal cells [59, 191]. The similarity of morphological characteristics, such as long cytoplasmic processes and protein expression patterns, suggests that the ependymal cells in the CVOs and CC are likely tanycytes. However, it is necessary to determine whether these tanycytes will be classified into the α-, β-, or unknown type which are specified in the mediobasal hypothalamus.

**Fig. 4:**
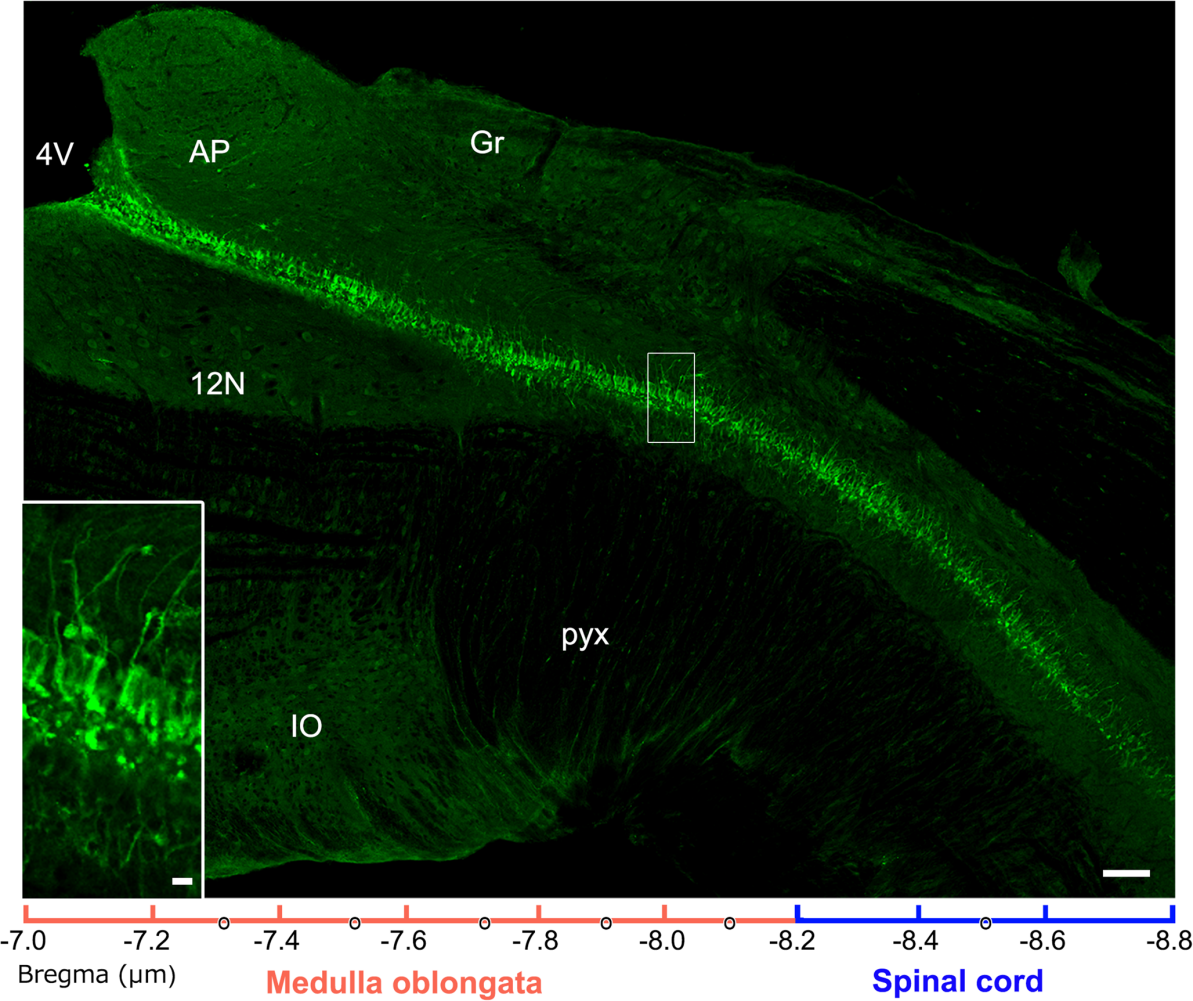
Sagittal section showing that EGFP-expressing tanycyte-like NSCs exist continuously along the CC from the medulla oblongata to spinal cord of adult mouse using Nestin-CreERT2/CAG-CAT^loxP/loxP^-EGFP transgenic mice. Scale bar = 50 (**A**) and 10 (**C**) µm. Photographs are rearranged from Scientific Reports [85]

In the adult mouse medulla oblongata, astrocyte-like NSCs are present in the AP, whereas tanycyte-like NSCs continuously exist along the CC [84, 86, 177] (Fig. 4). In the OVLT and SFO, similarly, tanycyte-like NSCs are present facing the third ventricle and astrocyte-like NSCs occur in the brain parenchyma [84, 86]. Tanycyte-like NSCs can significantly proliferate in response to EGF and FGF2 in vitro and in vivo [84, 177]. Tanycytes in the CC of the spinal cord are NSCs based on in vivo and in vitro proliferation experiments and marker protein characteristics [153]. Tanycytes express well-organized, tight junction proteins around the cell body, which may serve as a functional diffusion barrier [135, 169]. On the other hand, tanycytes in the CC import bioactive molecules from the CSF and vice versa export them into the CSF by transcytosis, suggesting that long cellular processes of tanycytes act as routes for communication between the CSF and brain parenchyma [182, 203, 205].

The neuroepithelial cells divide symmetrically to increase their number, expanding into the lateral telencephalon during early embryogenesis [197]. However, later, they transform into apical radial glia and produce mainly basal progenitors or, to a lesser extent, neurons of the developing neocortex [131]. Apical radial glia remains attached to the ventricular surface throughout the cell cycle, while the nucleus moves in the apical direction to the ventricular surface, and mitosis takes place during S-phase [239]. Since brain ventricular size is limited by the available space for mitosis of radial glia, this interkinetic movement is necessary for positioning the nucleus close to the centrosome and primary cilium [252]. The SVZ contains proliferative cells that are no longer attached to the ventricular surface and can divide anywhere within the SVZ to overcome this spatial limitation [224]. Thus, tanycytes on the ventricular surface retain traces of radial glia during development and are considered residual radial glial cells in the adult mammalian brain [94].

In the adult brain, various factors in the CSF promote the proliferation of NSCs in the SVZ [221]. The presence of mitogens is reported in the CSF of mice and humans: growth factors such as FGFs, VEGFs, and transforming growth factor-β; cell adhesion molecules such as L1 family of cell adhesion molecules, N-cadherin; axonal guidance molecules such as netrins, reelin, Slits, semaphorin, and ephrins [247]. The levels of exosomal miRNAs in the CSF control aging speed via hypothalamic NSCs [266]. The FGF17 in young CSF aims to restore oligodendrocyte proliferation and long-term memory consolidation in aged mice [115]. Therefore, the ventricular niche of tanycyte-like NSCs in the CC of the medulla oblongata and spinal cord is well-positioned to receive CSF-derived information to regulate proliferation and differentiation.

In addition, the cellular processes of α1-tanycytes are located close to or in contact with capillaries in the ventromedial nucleus of the hypothalamus, whereas those of α2‐tanycytes terminate on blood capillaries in the Arc [203]. Tanycytes are highly interconnected, specifically through connexin 43, to form a tight glial network with astrocytes, facilitating proliferative activity [198]. These results indicate that most ventricle-facing NSCs in the CC of the medulla oblongata and spinal cord, coordinately regulate their proliferation, based on information from both the fluid factors of the ventricles and the fenestrate capillary as in the SVZ and Arc.

In the CC of the mouse spinal cord, ependymal cells are morphologically classified into radial, cuboidal, and tanycytes [3, 101, 153]. They are also heterogeneous based on the expression of molecular markers [3, 153]. Ependymal cells generate mostly scar-forming astrocytes to prevent further damage and a few oligodendrocytes in mice after spinal cord injury, but new neurons are not generated [207, 210]. Recently, the neurosphere assay in vitro has demonstrated that cerebrospinal fluid-contacting neurons (CSF-cNs) express NSC markers, exhibit proliferative ability, and have potential for differentiation into neurons and oligodendrocytes [40]. Furthermore, spinal cord injuries or the injection of neurotrophic factors such as bFGF and VEGF into the lateral ventricle can enhance the proliferation of CSF-cNs [40]. Additionally, CSF-cNs are abundantly present in the spinal cord of a primate, the Rhesus monkey [124]. The NSC potential of some ependymal cell subtypes has been demonstrated, but whether ependymal cell diversity reflects different functions and regenerative capabilities remains unknown [163, 211, 228]. Since the CC is continuous from the medulla oblongata to the spinal cord, further investigation is needed to determine whether ependymal cells or tanycytes of the medulla oblongata have the same or different properties and functions from those present in the spinal cord.

## Characterization of NSCs in the Medulla Oblongata

### Protein Expression Profile of NSCs and Progenitor Cells

#### Astrocyte-like NSCs

Astrocyte-like NSCs expressed GFAP and Nestin in the periventricular, central, and lateral zones of the adult mouse AP [86]. Moreover, Nestin expression is confirmed by Nestin-EGFP and Nestin-CreERT2/GAG-CAT^loxP/loxP^ transgenic mice [26, 84, 86]. The expression percentage of GFAP and Sox2 in Nestin-expressing astrocyte-like NSCs is 99.5 and 96.6%, respectively, whereas OPCs and NPCs did not express GFAP and Nestin [86]. Vimentin is highly expressed in astrocytes of the adult rodent AP [19, 26, 84, 86]. Sox2 expression was detected in most astrocyte-like NSCs and OPCs but rarely in NPCs. Math1 is present in the cell cytoplasm when quiescent, however it moves from the cytoplasm to the nuclei during differentiation or proliferation [37, 174]. In the AP of the adult mouse, Math1 is a specific NPC marker, and doublecortin expression is only detected in cultured neurosphere cells of the adult mouse AP [26, 86, 111]. In the SVZ, Sox2 is expressed in type-B NSCs and type-C transient amplifying NPCs. However, doublecortin-expressing type-A NPCs do not, indicating that doublecortin expression is region and cell-type-specific. These results indicate that the expression profiles of molecular marker proteins in astrocyte-like NCSs and progenitor cells are similar to those observed in the SVZ and SGZ [114]. However, some features differ from SVZ and SGZ; most GFAP-positive astrocyte-like NSCs express S100β [86], although S100β is generally expressed in mature astrocytes, but not in NSCs of the SVZ and SGZ [64, 77]. S100β can be detected in a subgroup of specific postmitotic astrocytes, and cultured astrocytes from S100β-deficient mice show enhanced Ca^2+^ transients in response to treatment with KCl or caffeine, suggesting that it plays a role in maintaining Ca^2+^ homeostasis [255]. The expression of polysialylated neural cell adhesion molecule, a marker of types 2a and 2b NPCs in the SGZ, is highly expressed in the DVC, and electrical stimulation of the vagal afferents significantly decreases its levels [32, 33].

Astrocyte-like NSCs in the AP express sensor proteins involved in various signal transductions, including the Na^+^ sensor Nax, the LPS receptor TLR4 [175, 249]. TRPV1 is involved in a transduction pathway that involves Ca^2+^ influx [42] and may be required for Ca^2+^ homeostasis maintenance [147]. Astrocytes of the adult mouse AP exhibit an elongated and close-together shape rather than typical star-like shapes and express NSC-associated protein expressions such as Nestin, GFAP, and Vimentin [164]. Therefore, in the adult mouse AP, typical astrocytes are not present, and all GFAP-positive cells are NSCs. Thus, astrocyte-like NSCs are not only a source of cells but also a multifunctional astrocyte that accepts blood-derived information and transmits it to the brain. They also function as a diffusion barrier for small molecules that can replace typical BBB.

#### Tanycyte-like NSCs

Tanycyte-like NSCs express an NSC marker, Nestin, in the CC of the adult mouse medulla oblongata [84, 86]. Nestin expression is also confirmed by Nestin-EGFP and Nestin-CreERT2/GAG-CAT^loxP/loxP^ transgenic mice [26, 84, 86]. These results indicate that the expression profiles of molecular marker proteins in tanycyte-like NSCs of the CC are similar to astrocyte-like NSCs and those of the SVZ and SGZ [114]. A subpopulation of tanycyte-like NSCs expresses Pax6 in the CC, but Pax6 is not detected in those of the OVLT, SFO, and Arc [84]. Approximately 34% of ependymal cells express Nestin, and more than 90% of BrdU-labeled cells express Pax6, and heterozygotes of Pax6-deficient mice have been reported to have a much thinner granule cell layer and thinner GFAP^+^ radial glial cell projections [184]. Thus, the tanycyte-like ependymal cells of the adult CC express typical and common NSC marker proteins as found in B-type NSCs of the SVZ and type 1 NSCs of the SGZ.

Dorsally oriented ependymal cells possess well-developed microvilli and ZO-1-positive tight junctions in the spinal cord, but ventrally oriented ependymal cells do not [233, 237]. Apart from tanycyte-like NSCs, neurons in contact with the cerebrospinal fluid are present around the CC in the adult mouse spinal cord and medulla oblongata [40, 120]. These neurons, located within or below the ependymal cell layer, known as the stem cell niche, present a characteristic morphology with a dendrite projecting to the CC and ending with a protrusion that might serve to sense modification in the composition of either CSF or interstitial fluid [40, 120].

### Characteristics of Proliferation of NSCs and Progenitor Cells

#### Proliferation of NSCs

**Fig. 5:**
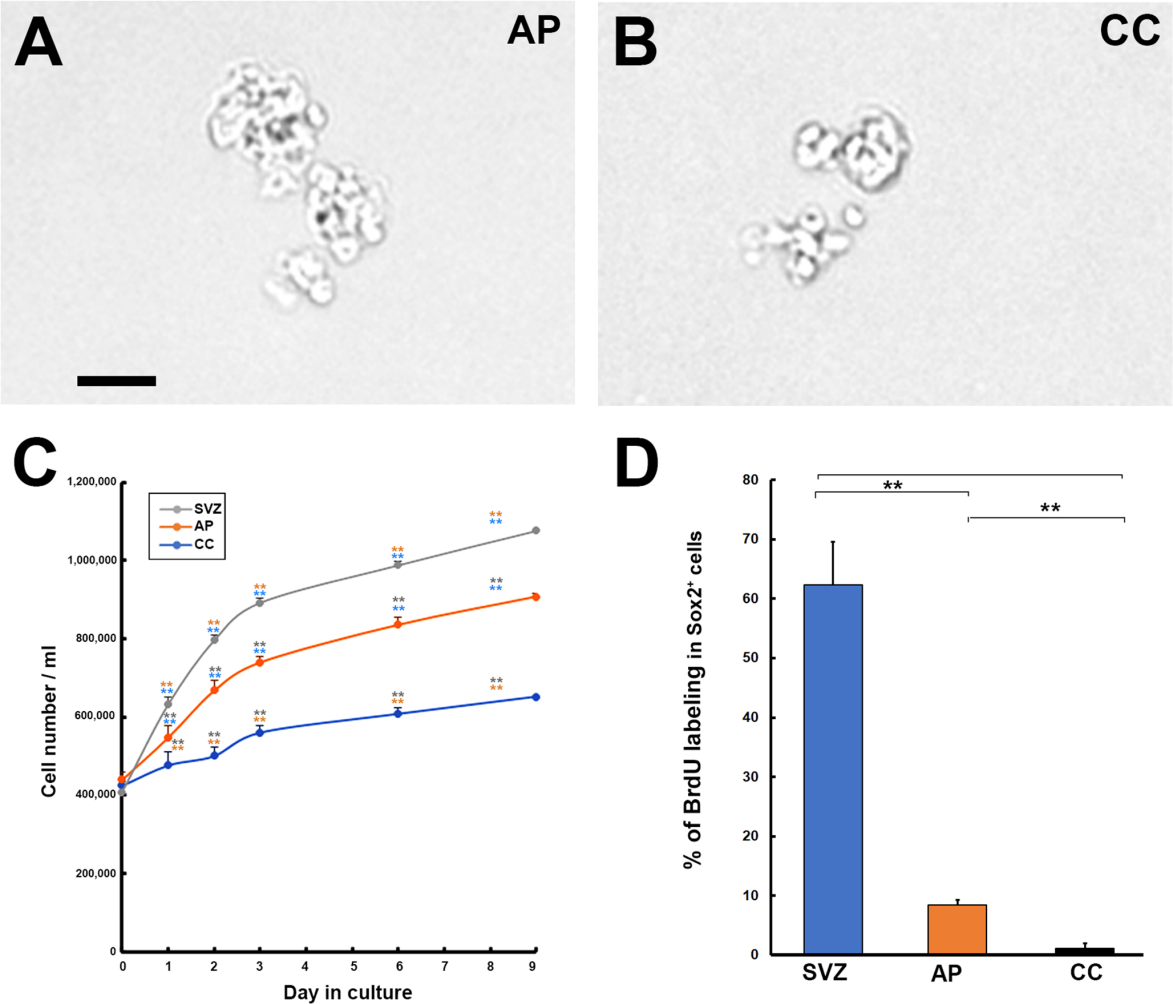
Phase microscopic images reveal the neurosphere from the AP (**A**) and CC (**B**) of the adult mouse brain after the sixth passage. Scale bar = 100 μm. Different proliferation of NSCs among the SVG, AP, and CC of adult mouse brain using neurosphere cells in vitro (**C**) and BrdU immunohistochemistry (**D**). Photographs are rearranged with permission from Elsevier [176]

Significant differences have also been found regarding the proliferation of NSCs of the AP and CC in the adult medulla oblongata. Proliferation experiments using neurospheres show that the proliferation rate is lower in tanycyte-like NSCs of the CC than in astrocyte-like NSCs of the AP in the presence of FGF-2 and EGF [177] (Fig. 5). In fact, in vivo studies using BrdU immunohistochemistry reveal that astrocyte-like NSCs in the AP moderately proliferate under normal conditions, but tanycyte-like NSCs in the CC are almost quiescent [177]. Furthermore, chronic treatment with AraC, which exhibits proliferating cell-specific toxicity, significantly reduced the number of astrocyte-likeNSCs, whereas the number of tanycyte-likeNSCs was not reduced [177]. The proliferation of NSCs in the mediobasal hypothalamus is much lower than that seen in well-characterized neurogenic zones in the adult brain, such as the SVZ or SGZ [136]. NSCs derived from the CC of the adult spinal cord are shown to be quiescent in vivo under physiologically normal conditions; however, NSCs at the dorsal tip of the CC proliferate symmetrically to maintain the ependymal layer [18]. In the SVZ, type-B NSCs are quiescent and dormant NSCs that do not divide. In contrast, quiescent type-B NSCs shift to highly proliferative type-C transiently amplifying NPCs, which differentiate sequentially into neuroblasts and immature neurons [44, 130].

Astrocyte-like NSCs of the AP express leptin receptors and leptin promotes their proliferation in vitro, whereas tanycyte-like NSCs do not express the receptors, and leptin does not affect proliferation [177]. Leptin-induced proliferation of astrocyte-like NSCs is observed when mice receive a 4-day leptin treatment in vivo [177]. Leptin has been shown to promote the proliferation of neurosphere cells from the embryonic cortex, possibly via the PI3K-Akt pathway [240]. Chronic administration of amylin, a satiating hormone, for 5 weeks activates NeuroD1 expression, increases the number of newly proliferated cells, and promotes their differentiation predominantly into neurons in the adult mouse AP [139]. On the other hand, food deprivation essentially decreases the proliferation of astrocyte-like NSCs in the adult mouse AP [177]. A possible explanation is that new neurons are required to control energy homeostasis and may be responsible for finely controlling food intake.

Cultured NSCs from the adult mouse AP express transient receptor potential cation channel subfamily C member 1 and Orai exhibit store-operated Ca^2+^ entries. Moreover, pharmacological inhibition of store-operated Ca^2+^ entries leads to decreased NSC proliferation and self-renewal, suggesting a significant role for store-operated Ca^2+^ entries in maintaining NSC proliferative activity [24]. Transient receptor potential cation channel subfamily C member 1 has diverse roles in the brain, such as the control of neurite outgrowth and axon guidance, regulation of neural progenitor cell proliferation and differentiation, regulation of neuronal apoptosis or survival, modulation of neuronal excitability and excitotoxicity [63].

#### Differentiation of NSCs

The proliferation of NPCs and OPCs in the AP is much higher than that of NSCs [84, 86]. This result is consistent with reports showing that NSCs proliferate slowly and NPCs divide actively in the SVZ and SGZ [62]. Thus, NSCs, OPCs, and NPCs in the medulla oblongata appear to have similar proliferative characteristics to those in the SVZ and SGZ; however, in the AP, typical astrocytes are absent, as all GFAP-positive cells are Nestin- and Vimentin-expressing NSCs. As for determining the fate of proliferating cells in the medulla oblongata under normal conditions, BrdU-labeled nuclei are observed abundantly in GFAP-expressing astrocyte-like NSCs and a few in mature oligodendrocytes and neurons in the AP, indicating that proliferation occurs mainly for self-neural of astrocyte-like NSCs [86]. In contrast, in the Sol and the dorsal motor nucleus of the vagus nerve, BrdU-labeled nuclei are seen mainly in oligodendrocytes, suggesting that oligodendrogenesis occurs from NSCs and OPCs [86]. On the other hand, when the differentiation of NCSs in cultured neurospheres was examined, more than 80% and about 10% of NSCs differentiated into astrocytes and oligodendrocytes, respectively [177]. These results indicate that the primary cell type differentiating from NSCs is glial cells rather than neural cells, unlike NSCs in the SVZ and SGZ.

### Environmental Factors Affecting the Proliferation and Differentiation of NSCs/NPCs

Intrinsic factors such as stressful stimuli [66], aging [90], and neuroinflammation [23] and lifestyle factors such as high-fat diets and alcohol [190] decline adult neurogenesis in the SGZ and SVZ. In contrast, physical exercise [69, 244] and antidepressant drug treatment [67, 148] have been shown to facilitate neurogenesis in adult mammalian brains. However, a few references concerning environmental factors that affect the proliferation and differentiation of NSCs and NPCs in the medulla oblongata have been reported, and therefore, the present review will summarize advances in these events.

Chronic running significantly increased the proliferation and survival of NPCs in the SGZ without changing the proliferation of NSCs [133, 243]. Chronic running leads to a cell cycle shortening of NeuroD1-expressing NPCs, but that of NSCs is not changed [75]. In contrast to the SGZ, chronic running significantly reduces the proliferation of astrocyte-like NSCs in the AP [177]. Vagotomy transiently increases the proliferating cells in the DVC of the adult rat [46]. The DVC is important in receiving cardiovascular, respiratory, gustatory, and orotactile information [216]. Suppression of NSC proliferation with chronic running may be related to running-induced alteration of visceral activity.

Our study has revealed that leptin receptor expression is detected in astrocyte-like NSCs, and the chronic leptin treatment augments the proliferation of astrocyte-like NSCs in the adult mouse AP in vivo and in vitro without affecting the differentiation of astrocyte-like NSCs [176]. Moreover, food deprivation remarkably declines the proliferation of astrocyte-like NSCs in the AP [176]. Leptin promotes the proliferation of NSCs derived from neurospheres of the embryonic cortex, possibly via the PI3K-Akt pathway [240]. NSCs and NPCs in the adult mouse AP respond more strongly to a short-term high-fat diet (HFD) than to a long-term one [87]. Long-term HFD increases astrocyte density in the Sol and 10 N and increases microglial and macrophage density in the AP and Sol, possibly by mild inflammation [87]. The knockdown of leptin receptors with shRNA in the AP and Sol promotes weight gain through increased fat mass and food intake [103]. These results agree with the previous study that switching to HFD induced rapid and transient increases in cell proliferation and neurogenesis in the Arc [96]. Activation of pituitary adenylate cyclase-activating polypeptide 1-expressing neurons of the NTS-AP causes sickness responses with decreased food and water intake and locomotor activity, and inhibition of them significantly attenuates these sickness responses [113]. Thus, these results indicate that the proliferation of astrocyte-like NSCs in the AP coordinately regulates energy homeostasis similar to that of the Arc.

Chronic corticosterone treatment significantly decreased the proliferation of astrocyte-like NSCs in the adult mouse AP [176]. This result aligns with the study, which found that chronic immobilization stress decreases the number of proliferating cells and mature neurons in the AP [49]. In the SGZ, chronic corticosterone treatment decreases the number of immature neurons without changing the total number of proliferating cells [48]. Immobilization stress and corticosterone treatment increase oligodendrogenesis and decrease neurogenesis without altering the proliferation of NSCs in the SGZ [48]. Since NSCs and progenitor cells in the SGZ are actively dividing, it is possible to change the phenotype of differentiated mature cells by adjusting the number of surviving progenitor cells through apoptosis [116]. On the other hand, in the AP, tanycyte-like NSCs are almost quiescent, and astrocyte-like NSCs and progenitor cells proliferate slowly. Therefore, in the AP, increased differentiation from NSCs and proliferation of progenitor cells are more efficient ways to control mature cells in response to environmental alterations.

## Neurogenesis and Oligodendrogenesis in the Adult Medulla Oblongata

### Neurogenesis in the Adult Medulla Oblongata

The unilateral cervical vagotomy, which alters vagal modulation of autonomic function, drastically increases proliferating cells in the DVC, consisting of the AP, Sol, and the dorsal motor nucleus of the vagus nerve of the medulla oblongata [21]. A study of traumatic injury with a controlled cortical impact significantly enhanced the proliferation of astrocytic NSCs and NPCs in the AP [73]. Our previous study reported that medullary hemorrhage induced by intramedullary administration of collagenase promotes a marked increase of astrocyte-like and tanycyte-like NSC proliferation [84]. Ependymal cells in the spinal cord reveal restricted responses to the injured segment and are absent in adjacent segments after spinal cord injury [91, 153]. NPCs can become activated, proliferate, migrate to the injury sites, and differentiate into mature neural cells, but NPC activation is insufficient to induce adequate structural repair or functional recovery in the spinal cord [91].

**Fig. 6:**
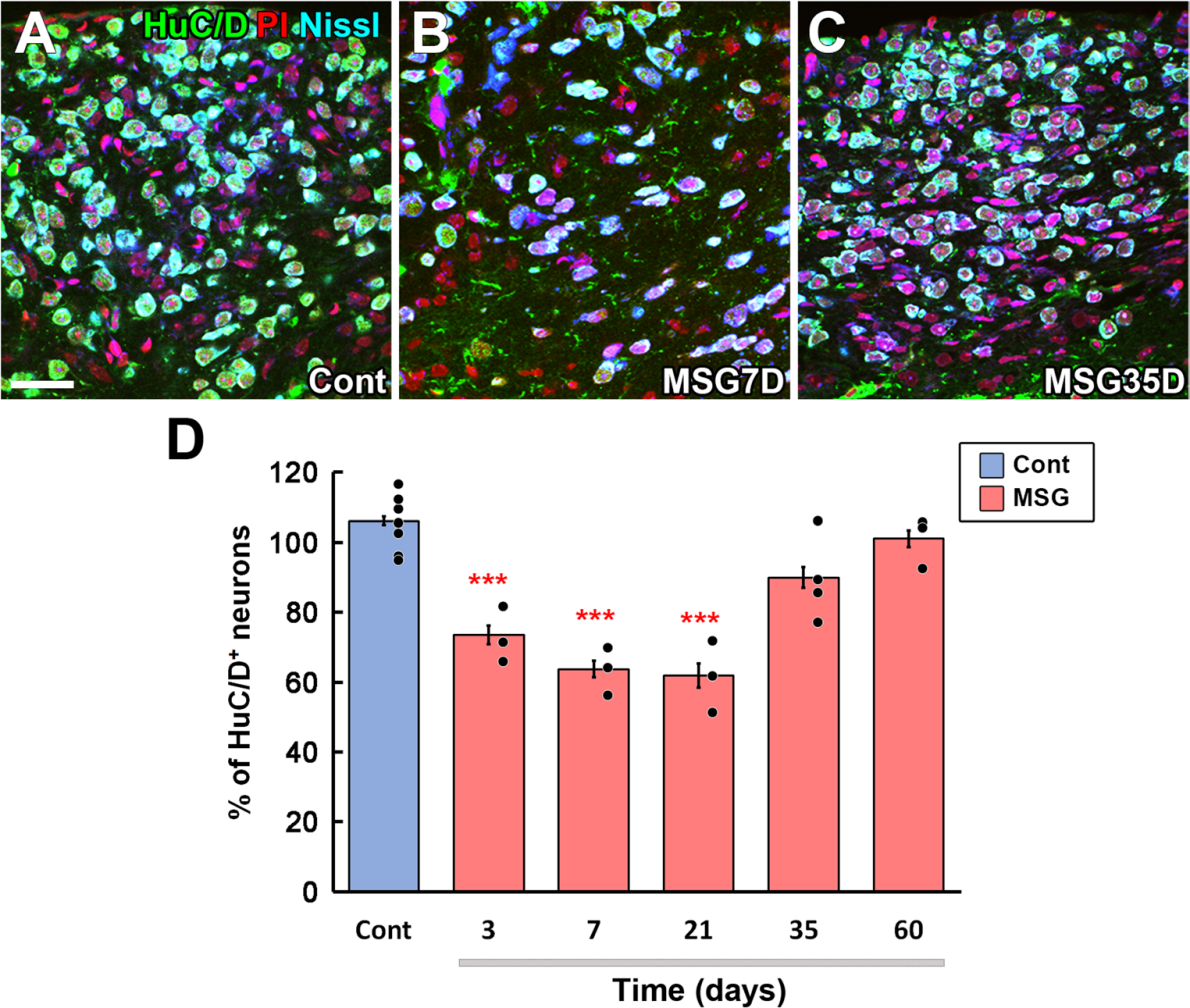
Confocal microscopic images showing changes in the neuronal density of the adult mouse AP 7 and 35 days after high-dose subcutaneous administration of MSG (**A-C**). The quantitative morphometrical analysis revealing a significant decline of neuronal density 3, 7, and 21 days after MSG treatment, but its density returns to normal 35 days (**B**). Scale bar = 50 μm. *** *P* < 0.001 vs. control with One-way ANOVA with Tukey’s post hoc test. Photographs are rearranged with permission from Elsevier [82]

**Fig. 7:**
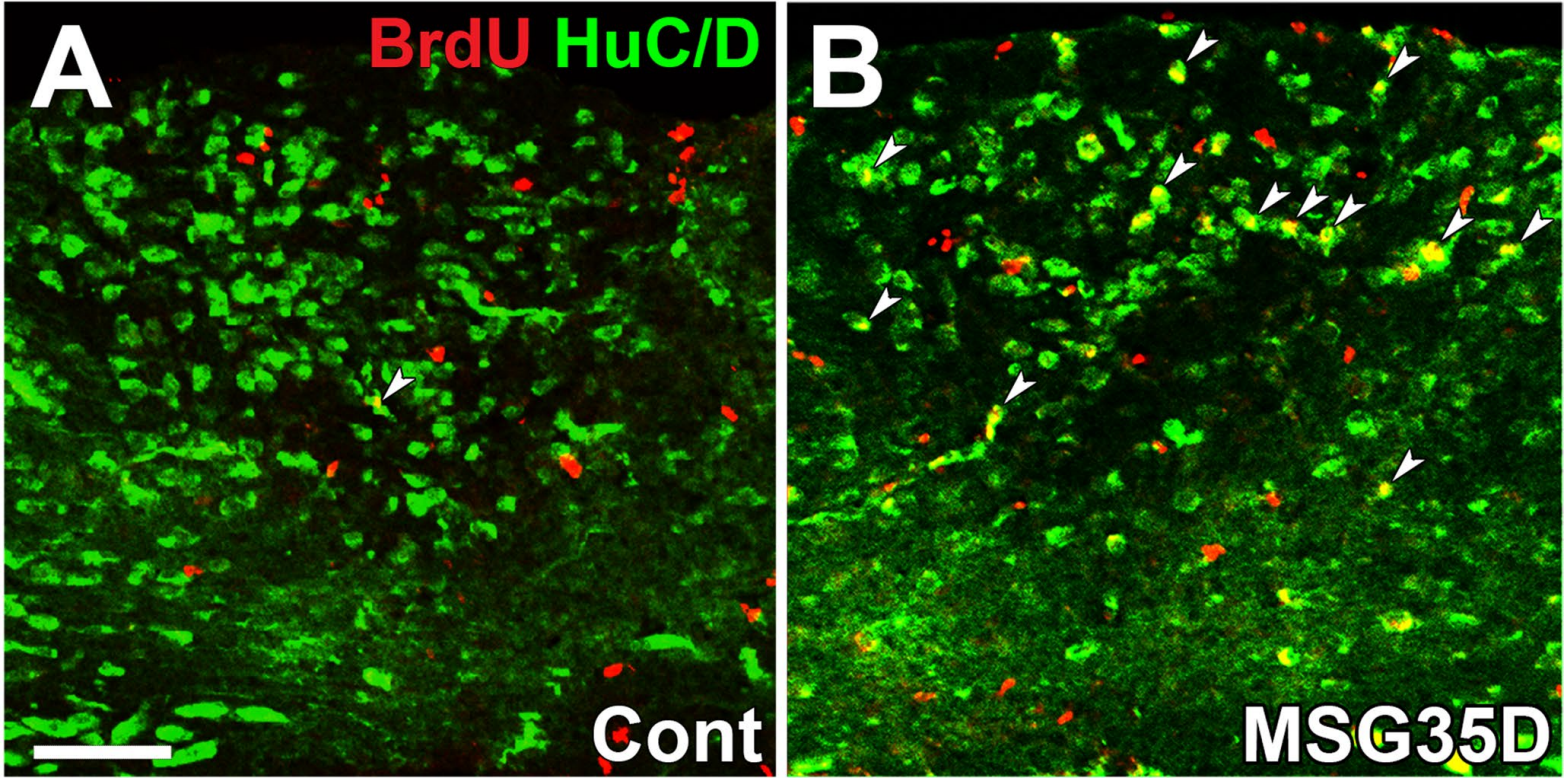
Confocal microscopic images showing neurogenesis in the adult mouse AP 35 days after MSG treatment using BrdU and HuC/D immunohistochemistry. Scale bar = 50 μm. Photographs are rearranged with permission from Elsevier [82]

Glutamate is thought to circulate systemically through the bloodstream. It cannot reach BBB-containing brain regions, whereas the AP lacks the typical BBB, and therefore, substances with molecular weights below 5,000 easily reach the AP parenchyma [164, 166, 181]. Chronic intake of MSG is reported to cause metabolic syndrome or obesity by brain inflammation and leptin resistance, possibly by attacking BBB-lacking brain regions [10]. Our study shows that peripheral MSG administration induces a remarkable increase in Fos expression in the adult mouse brain AP [82]. This acute neuronal activation results in a prominent increase in neuronal cell death and, vice versa, a decrease in density in HuC/D-expressing neurons [82]. MSG administration elevates glutamate levels in the AP of young mice without a significant increase in other brain regions [193]. The administration of MSG significantly increases the density of microglia and phagocytic microglia with morphological conversion to amoeboid shape, and activated microglia rapidly surround dying neurons after MSG treatment in adult mice [82]. ATP is concerned with a find-me signal for dying and/or dead cells with microglial processes elongation to the brain injury site [251]. Interestingly, 40% of neurons in the adult mouse AP are lost within in a few days after MSG treatment, but nearly complete recovery of neuronal density occurs by day 35 [82] (Fig. 6). The proliferation of NSCs and NPCs is significantly increased just after the MSG treatment and BrdU-incorporated mature neurons appear several weeks after the treatment [82] (Fig. 7). These results indicate that proliferating cells in the adult AP are largely concerned with complete regeneration of neuronal density.

### Oligodendrogenesis in the Adult Medulla Oblongata

There have been numerous reports of neuronal and glial damage in experimental animal models and human clinical reports for demyelinating diseases, including multiple sclerosis (MS) and amyotrophic lateral sclerosis [36, 54]. MS is shown to be a heterogeneous disease by magnetic resonance imaging (MRI) analysis, and lesions in the medulla oblongata are standard in MS patients [194, 200]. Lesions in the lower olives of the medulla oblongata are observed primarily in the acute phase of rats with EAE, an animal model of MS. In contrast, lesions in the cerebellum and spinal cord are observed in the chronic phase [50]. Furthermore, MRI analysis has revealed that a decrease in the volume of the medulla oblongata is associated with the degree of spinal cord damage in MS patients [141]. In rodents, many immune cells are observed in the CVOs, including the AP of EAE-induced animals [217]. Therefore, inflammation and subsequent damage are expected in the medulla oblongata of EAE model animals.

**Fig. 8:**
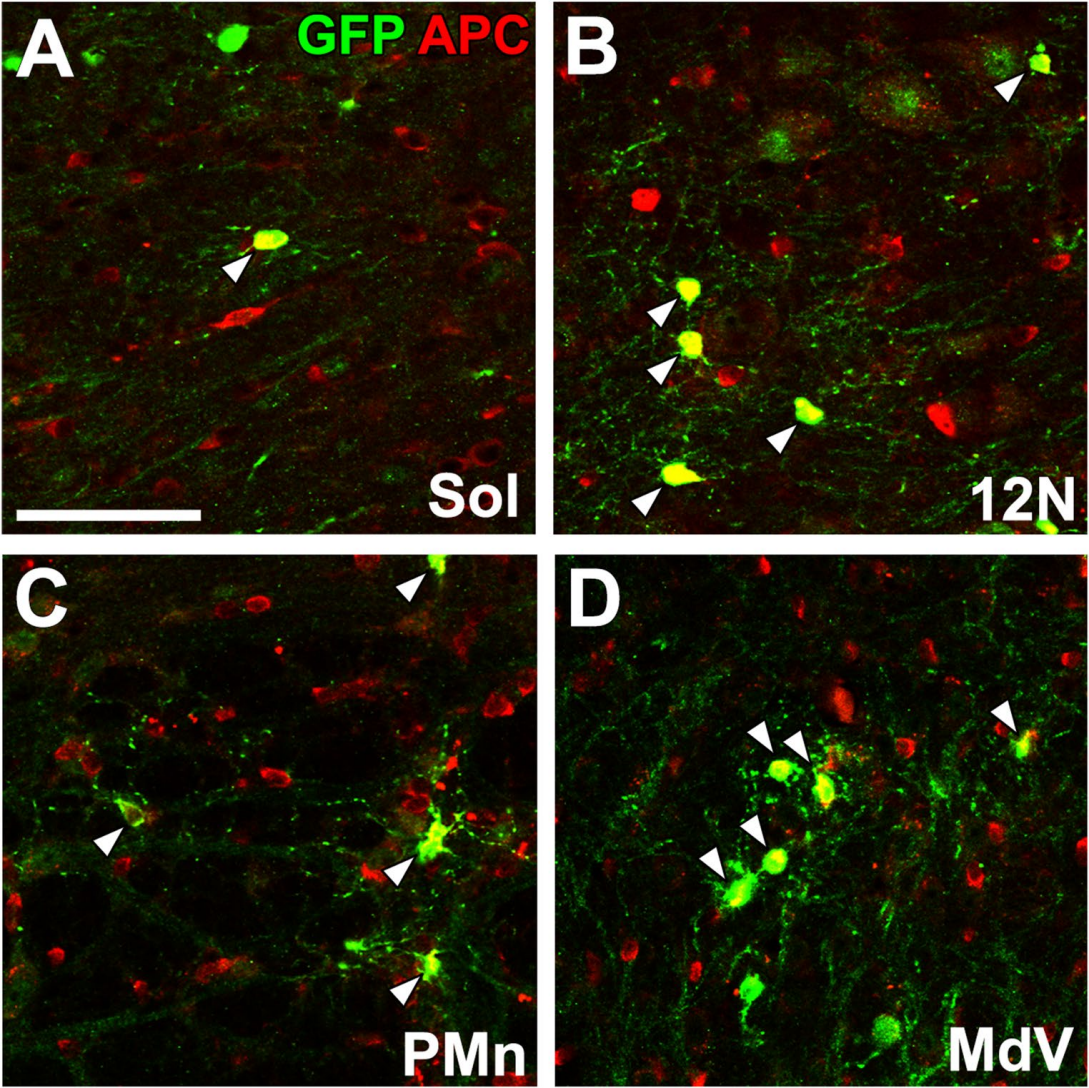
Confocal microscopic images revealing oligodendrogeneis in the adult mouse 50 days after EAE. using Nestin-CreERT2/CAG-CAT^loxP/loxP^-EGFP transgenic mice Scale bar = 50 μm. Photographs are rearranged with permission from Elsevier [105]

Our previous study with EAE-induced mice revealed enhanced differentiation into mature oligodendrocytes in various medullary regions originating from NSCs and resident oligodendrocyte progenitor cells [105] (Fig. 8). Localized demyelination by lysophosphatidylcholine (LPC) shows a dramatic increase in the proliferation of resident oligodendrocyte progenitor cells (OPCs) in the medulla oblongata, but little contribution of NSCs is reported to concern remyelination after LPC-induced localized demyelination [106]. This is because LPC-induced demyelination injuries are more minor in area and shorter in duration than EAE-induced demyelination injuries, allowing resident OPCs to provide an adequate supply of cells without depending on NSCs. In contrast, NSCs supply oligodendrocytes to the corpus callosum and striatum after LPC-induced focal demyelination in the SVZ [41, 155]. Thus, these results indicate that NSCs contribute to severe demyelination injury of the adult medulla oblongata.

## Perspective of Future Survey

Brain injuries such as stroke, trauma, and some neurodegenerative conditions can activate endogenous NSCs and NPCs [104, 110]; however, this activation is not universal (e.g., adult hippocampal neurogenesis is attenuated in Alzheimer’s disease) [187, 270]. Nevertheless, this activation is not universal across diseases. SVZ-derived NPCs are unlikely to reach damaged sites of the cerebral cortex upon injury, and cannot survive without functional maturation and integration into the local neural circuit [122, 263]. SGZ-derived NPCs migrate to mislocate at the hippocampal outer layer across the boundary of the inner granular cell layer after severe traumatic brain injury [112]. These higher-function centers have relatively low regenerative capability probably owing to a strategy to avoid memory malfunction for higher-order brain functions. The MBH (a diencephalic region) and the medulla oblongata (a brainstem structure) are evolutionarily conserved and control fundamental homeostatic functions. Despite their stereotyped circuitry, the MBH exhibits substantial plasticity—including synaptic remodeling, activity-dependent gliogenesis/oligodendrogenesis, and state- or injury-induced neurogenesis. These neuronal networks may show context-dependent regenerative responses in rodents, unlike higher-order associative cortices. Recent evidence indicates that near-complete restoration of AgRP neuron density occurs in the adult mouse Arc [261], and an alternative hypothalamic-neurohypophysial system is reconstructed in the adult mouse ME [185]. In the adult medulla oblongata, similarly, intrinsic NSCs and progenitor cells of AP are also activated by neuronal elimination with MSG, and neuronal density returns toward baseline after MSG treatment. Moreover, proliferating progenitor cells and NSC-derived OPCs are responsible for regenerating oligodendrocytes of various medullary regions. Currently, treatments for brain injury focus on pharmacological, rehabilitation training, and cell-based therapies [232]. The present review suggests that NSCs and progenitors in the MBH (diencephalon) and medulla oblongata (brainstem) have preclinical, context-dependent therapeutic potential for the endogenous repair response of damaged adult subcortical regions. Direct demonstrations of regenerative replacement in humans are currently sparse, particularly in the brainstem/medulla where evidence is lacking. Our conclusions for the brainstem therefore rely on rodent data. In contrast, the MBH has supportive human evidence, including NSC/tanycyte markers and injury-associated proliferative responses in the Arc and ME, suggesting conserved plasticity.

In addition to in vivo models, emerging human iPSC-derived organoid and assembloid systems provide a translational platform to test these hypotheses in a human genetic background. Region-patterned organoids that approximate hypothalamus, midbrain, and hindbrain features can recapitulate key transcriptional programs, gliogenic lineages, and niche signals, enabling controlled perturbation of Notch/BMP/Wnt and inflammatory pathways. Patient-derived iPSCs further allow modeling disease-specific constraints on neurogenesis and myelination and evaluation of pro-regenerative interventions in a human context [119, 134, 188, 195]. Accordingly, future strategies will likely require mitigation of inflammation-induced growth-inhibitory niche signals, including IL-1β/tumor necrosis factor-α/IL-6/interferon-γ pathways and glial-scar extracellular matrix, together with rebalancing Notch/BMP/Wnt pathways to unlock endogenous repair [23, 44, 54, 187, 207, 209, 221].

## Data Availability

No datasets were generated or analysed during the current study.
